# G3BP1 promotes tumor progression and metastasis through IL-6/G3BP1/STAT3 signaling axis in renal cell carcinomas

**DOI:** 10.1038/s41419-018-0504-2

**Published:** 2018-05-02

**Authors:** Yong Wang, Donghe Fu, Yajing Chen, Jing Su, Yiting Wang, Xin Li, Wei Zhai, Yuanjie Niu, Dan Yue, Hua Geng

**Affiliations:** 1 Department of Urology, The Second Hospital of Tianjin Medical University, Tianjin Institute of Urology, Tianjin Medical University, Tianjin, 300211 China; 2 Department of Microbiology, School of Medical Laboratory, Tianjin Medical University, Tianjin, 300203 China; 3 Research Center of Molecular Biology, Inner Mongolia Medical University, Hohhot, 010059 China; 4 Research Center of Basic Medical Sciences, Tianjin Medical University, Tianjin, 300070 China; 5 Department of Pharmacology, Tianjin Medical University, Tianjin, 300070 China; 6 Department of Urology, Renji Hospital, School of Medicine in Shanghai Jiao Tong University, Shanghai, 200127 China

## Abstract

The chronic inflammatory microenvironment within or surrounding the primary renal cell carcinoma (RCC) site promotes oncogenic transformation as well as contributes to the development of metastasis. G3BP stress granule assembly factor 1 (G3BP1) was found to be involved in the regulation of multiple cellular functions. However, its functions in RCC have not been previously explored. Here, we first showed that the expression of G3BP1 is elevated in human RCC and correlates with RCC progression. In cultured RCC cells, knockdown of G3BP1 results in inhibition of tumor cell proliferation, migration, and invasion, consistently with the alteration of epithelial–mesenchymal transition (EMT) and cell proliferative markers, including Cadherins, Vimentin, Snail, Slug, c-Myc, and cyclin D1. Remarkably, knockdown of G3BP1 dramatically impaired the signaling connection of pro-inflammatory cytokine IL-6 stimulation and downstream STAT3 activation in RCC, thus eventually contributing to the disruption of IL-6-elicited RCC migration and metastasis. In addition, *in vivo* orthotopic tumor xenografts results confirmed that knockdown of G3BP1 suppressed RCC tumor growth and metastasis in mice. Collectively, our findings support the notion that G3BP1 promotes tumor progression and metastasis through IL-6/G3BP1/STAT3 signaling axis in RCC.

## Introduction

Renal cell carcinoma (RCC) is the most common solid cancer of the adult kidney and accounts for ~90% of kidney neoplasms^1^. More than 350,000 people are diagnosed with renal cell cancer worldwide, and an estimated 140,000 people die from the disease each year^2^. Many cases of RCC are asymptomatic until the condition becomes malignant. As a result, local invasion or metastatic disease is already present in about one-third of cases at the time of diagnosis^3^. Clear cell RCC is the most prevalent subtype of RCC. Its characteristic high metastatic potential and resistance to traditional radiotherapy and chemotherapy present a major challenge for managing the disease^3,4^. Although surgical intervention followed by immunotherapy has emerged a major therapeutic option for RCC with metastasis, it has failed to demonstrate clear benefits as a therapeutic strategy for the overall survival of RCC patients^3,5^. The identification of molecular targets modulating RCC progression and metastasis would provide useful information for tailoring targeted treatments for patients with advanced RCC^6^.

The chronic inflammatory microenvironment is implicated to trigger cellular events that induce oncogenic transformation of cells and distal metastasis^7,8^. Cytokines are pivotal players of the tumor microenvironment that may be contributing towards RCC pathogenesis. Interleukin 6 (IL-6) is one of the most studied cancer-associated cytokines, and elevated levels of IL-6 have been found in primary RCC cultures, RCC cell lines, as well as in the serum from RCC patients^9–12^. Primarily, IL-6 activates signal transducer and activator of transcription 3 (STAT3) signaling thus promotes tumor cell proliferation and enhances cell invasiveness in cancers, which is in line with the constitutive activation of STAT3 in RCC, especially in metastatic disease^13,14^. Recently, blockade of the IL-6/STAT3 pathway was considered as a potential therapeutic approach for RCC treatment^15–17^. Thus, fully understanding the role and mechanism of IL-6/STAT3 signaling in RCC metastasis will be important for uncovering the novel molecular targets for RCC immunotherapy.

G3BP stress granule assembly factor 1 (G3BP1, also known as GTPase-activating protein SH3 domain-binding protein 1), is an RNA-binding protein involved in the regulation of multiple cellular functions^18^. Previous studies showed that G3BP1 regulates mRNA stability in response to extracellular stimuli, and plays an important role in stress granule (SG) formation^19–22^. In addition to its RNA-binding activity, G3BP1 promotes S-phase entry and controls cell proliferation in fibroblast^23^. Furthermore, G3BP1 regulates cell apoptosis through interaction with p53 and affecting its cellular translocation^24,25^. More recently, the overexpression of G3BP1 has been implicated in human cancers, including breast, gastric, colon, and liver carcinomas, suggesting the oncogenic and functional role of G3BP1 in tumorigenesis^26–29^. However, it remains unknown whether and how G3BP1 contributes to RCC progression and metastasis.

In this report, we explored the expression of G3BP1 in primary RCC and its association with clinicopathological parameters. Functionally, we investigated the effects of G3BP1 on RCC cell proliferation, migration, and invasion *in vitro* and *in vivo*. We further defined the role of G3BP1 in linking inflammatory cytokine IL-6 with the activation of STAT3 signaling in RCC.

## Result

### G3BP1 is frequently upregulated in RCC patients

**Fig. 1:**
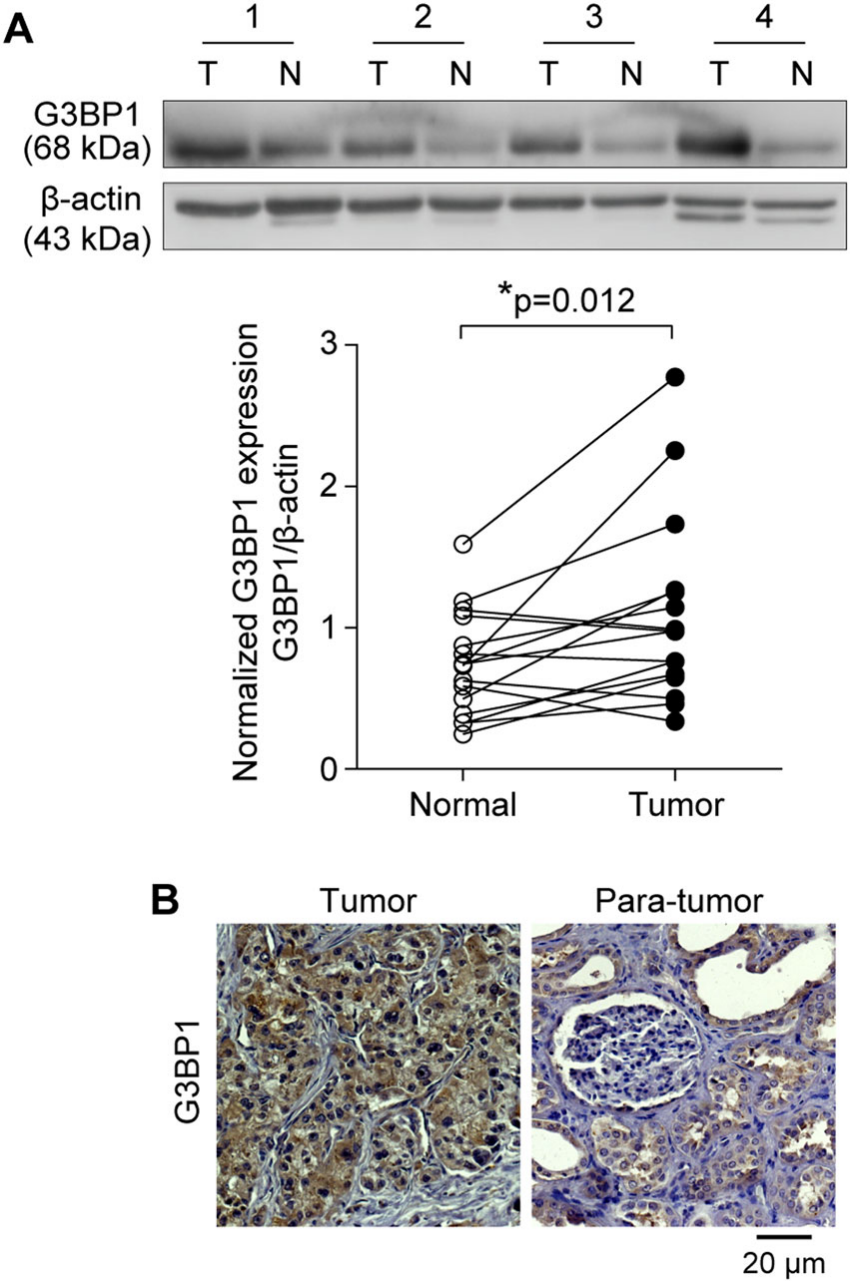
The expression of G3BP1 is increased in RCC. **a** G3BP1 expression in 16 pairs of primary RCC (T) and adjacent normal kidney tissues (N) were examined by Western blot. Upper panel: representative four pairs of Western blot images. Lower panel: the expression of G3BP1 was quantified by normalizing with β-actin, and all 16 pairs of RCC patient samples were analyzed using a paired *t*-test, *p* = 0.012. **b** G3BP1 expression was examined by immunohistochemistry staining in a study cohort with 43 pairs of RCC (tumor) and adjacent normal kidney tissues (para-tumor). Representative G3BP1 staining of RCC and paired non-tumor sections were shown, and brown signal indicated the G3BP1-positive staining.

We first examined the expressions of G3BP1 in the 16 pairs of RCC tissues and their corresponding adjacent normal kidney tissues by Western blotting. The results showed that G3BP1 expression was significantly (*p* = 0.012) increased in the RCC tissues when compared with adjacent non-cancerous kidney tissues (Fig. 1a). To further investigate the correlation of G3BP1 expression with RCC clinicopathologic features, a cohort comprising of 43 RCC patients were subjected to immunohistochemical (IHC) staining with an antibody specifically against G3BP1 (Fig. 1b). All slides were reviewed by two pathologists at double-blinded manner for IHC scoring based on the percentage and intensity of G3BP1 staining signals^30,31^. Consistent with Western blotting results, the expression level of G3BP1 was significantly higher in 51.2% (22/43) of the RCC samples, in contrast, G3BP1 expression was dramatically reduced to 25.6% (11/43) in para-tumor normal tissues (*p* = 0.0147, OR = 3.048, 95% CI 1.227–7.568), suggesting the expression level of G3BP1 in RCC tumors was markedly elevated as compared with the adjacent normal tissues. Further analysis revealed that the expression level of G3BP1 in RCC patients is significantly (*p* < 0.05) associated with higher levels of TNM stages and Fuhrman grade (Table 1). Meanwhile, no significant (*p* > 0.05) associations were identified between the expression of G3BP1 and any other clinicopathological characteristics, including patient’s age, gender, RCC subtype, tumor size, tumor side, and necrosis (Table 1). Taken together, our findings suggest that the expression of G3BP1 is increased in RCC and its expression level is associated with the progression of RCC.

**Table 1:** Correlation between G3BP1 expression and clinicopathologic characteristics in RCC patients

			G3BP1 expression		
		N	−	+	*p* Value
Gender	Male	29	12	17	0.159
	Female	14	9	5	
Age	<60	16	8	8	0.907
	≥60	27	13	14	
Tumor size	<4	6	4	2	0.188
	>4, ≤7	6	1	5	
	>7	31	16	15	
TNM stage	T1–2	25	20	5	0.000*
	T3–4	18	1	17	
Fuhrman grade	I, II	27	17	10	0.027*
	III, IV	16	4	12	
Location	Tumor	43	21	22	0.015*
	Para-tumor	43	32	11	
RCC subtype	ccRCC	38	19	19	1.000
	Non-ccRCC	5	2	3	
Tumor side	Left	19	12	7	0.095
	Right	24	9	15	
Necrosis	Negative	15	5	10	0.137
	Positive	28	16	12	

Note: G3BP1 expression (−/+) was determined by IHC staining score. The scoring system was composed of two independent parameters: percentage of tumor cells exhibiting positive staining (0–5: 0–1%; 1–5%; 6–10%; 11–20%; 21–50%; >50%); and intensity of IHC staining (0–2: 0, no staining; yellow-brown staining; strong brown staining). The final scores of two parameters were added to a sum and defined as the IHC staining score. 0–3: Negative; 4–7: Positive.

*p  < 0.05 indicates statistical significance by *χ*^2^ test.

### Silencing of G3BP1 represses the mesenchymal phenotype in RCC cells *in vitro*

**Fig. 2:**
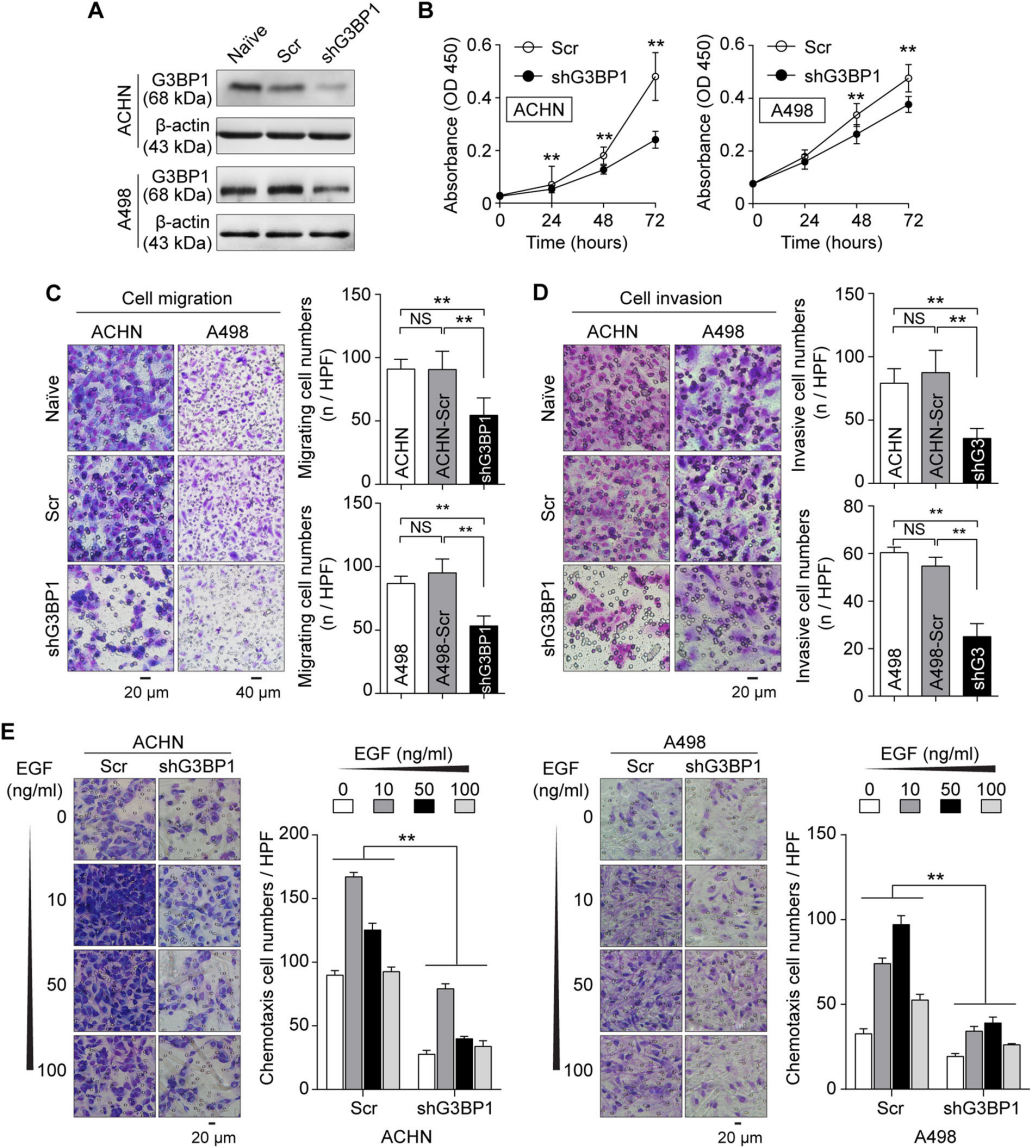
Knockdown of G3BP1 represses RCC mesenchymal phenotype *in vitro*. **a** ACHN and A498 cells were transduced with lentivirus-mediated G3BP1-specific shRNA (shG3BP1) or scramble control (Scr), and the efficiency of G3BP1 knockdown was examined by Western blot. **b** ACHN and A498 cells with G3BP1 knockdown (shG3BP1) or scramble control (Scr) were cultured in growth medium and cell proliferation was determined by CCK8 assay at indicated time points, **p < 0.01 by two-way ANOVA. **c** Transwell assay was performed to determine the cell migration. Left panel: representative microscopic images, Naïve: untreated; Scr: scramble control; shG3BP1: G3BP1 knockdown. Right panel: quantitative analysis of cell migration, data were presented as mean ± s.d., *n* = 10 (ACHN) and *n* = 6 (A498) respectively, **p < 0.01 by one-way ANOVA, NS: no significant difference. **d** Matrigel cell invasion assay was used to evaluate the cell invasive ability. Left panel: representative microscopic images, Naïve: untreated; Scr: scramble control; shG3: G3BP1 knockdown. Right panel: quantitative analysis of cell invasion, data were presented as mean ± s.d., *n* = 10 (ACHN) and *n* = 6 (A498) respectively, **p < 0.01 by one-way ANOVA, NS: no significant difference. **e** G3BP1 knockdown impaired EGF-induced chemotaxis. Scr: scramble control; shG3BP1: G3BP1 knockdown. Data were presented as mean ± s.d. from three independent repeats, **p < 0.01 by two-way ANOVA.

In order to investigate the role of G3BP1 in RCC progress and metastasis, two RCC cell lines (ACHN and A498) with different potential for metastasis were selected as *in vitro* cell models^32^. RCC cells with lentivirus-mediated G3BP1 stable knockdown were used for functional studies (Fig. 2a and Suppl Fig. 1). The efficiency of G3BP1 knockdown was confirmed at both mRNA and protein levels by quantification of qRT-PCR (Supplementary Fig. 1A) and Western blot (Suppl Fig. 1B), respectively. G3BP1 knockdown cells expressed <35% of detectable G3BP1 as compared to scramble control cells were qualified as G3BP1 knockdown and used for further experiments. The effects of G3BP1 on RCC cell proliferation was then examined using CCK8 assay. The results indicated downregulation of G3BP1 significantly (*p* < 0.01) inhibited cell proliferation in both ACHN and A498 cells (Fig. 2b). Further transwell assay showed that G3BP1 knockdown strongly reduced the migrated RCC cell number when compared to the scramble controls (Fig. 2c). Together, these data suggested that G3BP1 plays an important role in RCC cell proliferation and migration *in vitro*. Since enhanced tumor cell migratory capacity is one of the characteristics of tumor metastasis, further tests based on loss-of-function approach were used to examine the effects of G3BP1 on RCC tumor cell mesenchymal phenotypes. The results showed that G3BP1 downregulation led to the decrease of tumor cell invasion in the Matrigel invasion assay (Fig. 2d). Moreover, it was known that EGFR and its ligand EGF are frequently overexpressed in RCC, and its signaling cascade is critical for RCC metastasis^33–35^. Specifically, we found that EGF-induced chemotaxis, a pivotal event for RCC metastasis, was significantly (*p* < 0.01) impaired in G3BP1 knockdown cells when compared to controls as indicated by micro-Boyden chamber assays (Fig. 2e). Collectively, these results indicated that G3BP1 contributes to promoting RCC cell proliferative, migratory, and invasive capacities, thereby facilitating the transitioning of tumor mesenchymal phenotype.

### Knockdown of G3BP1 inhibited expressions of cell proliferative and EMT markers in RCC

**Fig. 3:**
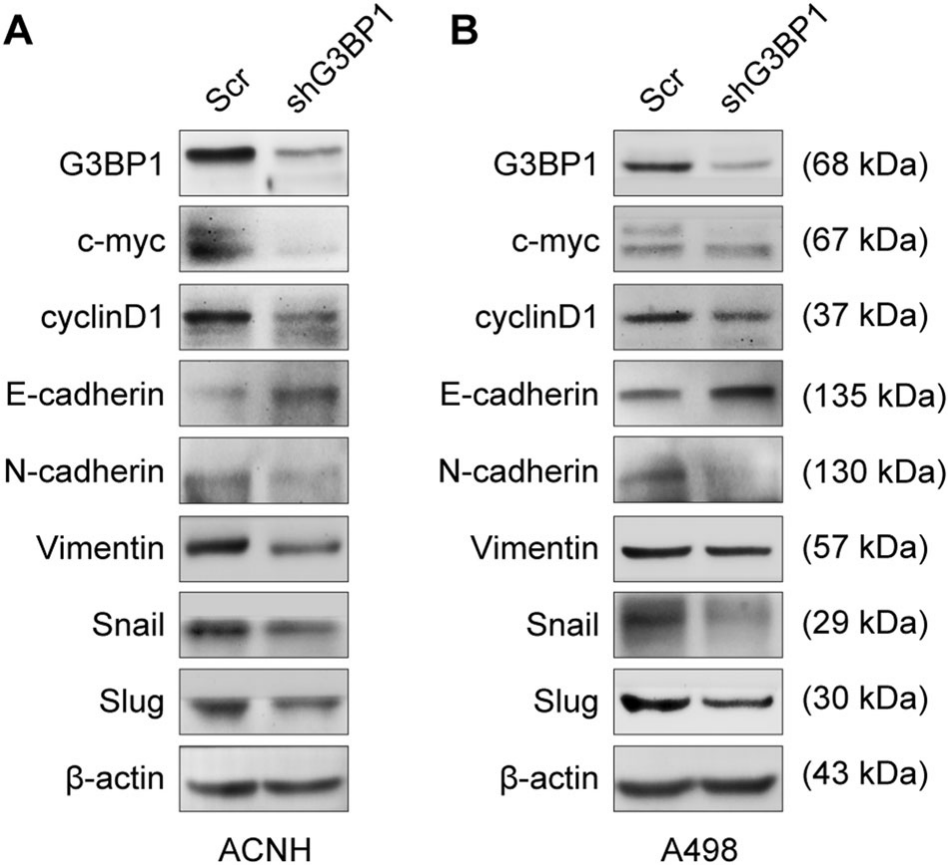
Knockdown of G3BP1 suppressed expressions of cell proliferative and EMT markers in RCC cells. ACHN (**a**) and A498 (**b**) cells were transduced with lentivirus-mediated G3BP1-specific shRNA (shG3BP1) or scramble control (Scr). Western blot analysis was conducted to determine the target molecule expressions of cell proliferative markers (c-Myc and cyclinD1) and EMT markers (E-cadherin, N-cadherin, vimentin, snail, and slug).

To further investigate the role of G3BP1 in RCC proliferation and metastasis, G3BP1 was knocked down in ACHN and A498 RCC cells, and the expression levels of cell proliferative and epithelial–mesenchymal transition (EMT) markers were examined by Western blot. As shown in Figure 3, the expressions of c-Myc and cyclin D1 were downregulated in G3BP1 knockdown RCC cells, consistent with the inhibition of cell proliferation in shG3BP1 cells (Figs. 2b, 3a, b). In addition, the expression of the epithelial cell–cell adhesion molecule E-cadherin was upregulated. Consistently, the expressions of mesenchymal markers N-cadherin, Vimentin, Snail, and Slug were dramatically decreased in G3BP1 knockdown cells (Fig. 3a, b). Thus, it is suggested that downregulation of G3BP1 suppresses not only cell proliferation but also EMT in RCC cells.

### Knockdown of G3BP1 impaired IL-6-induced STAT3 activation and led to suppression of RCC migration and invasion

**Fig. 4:**
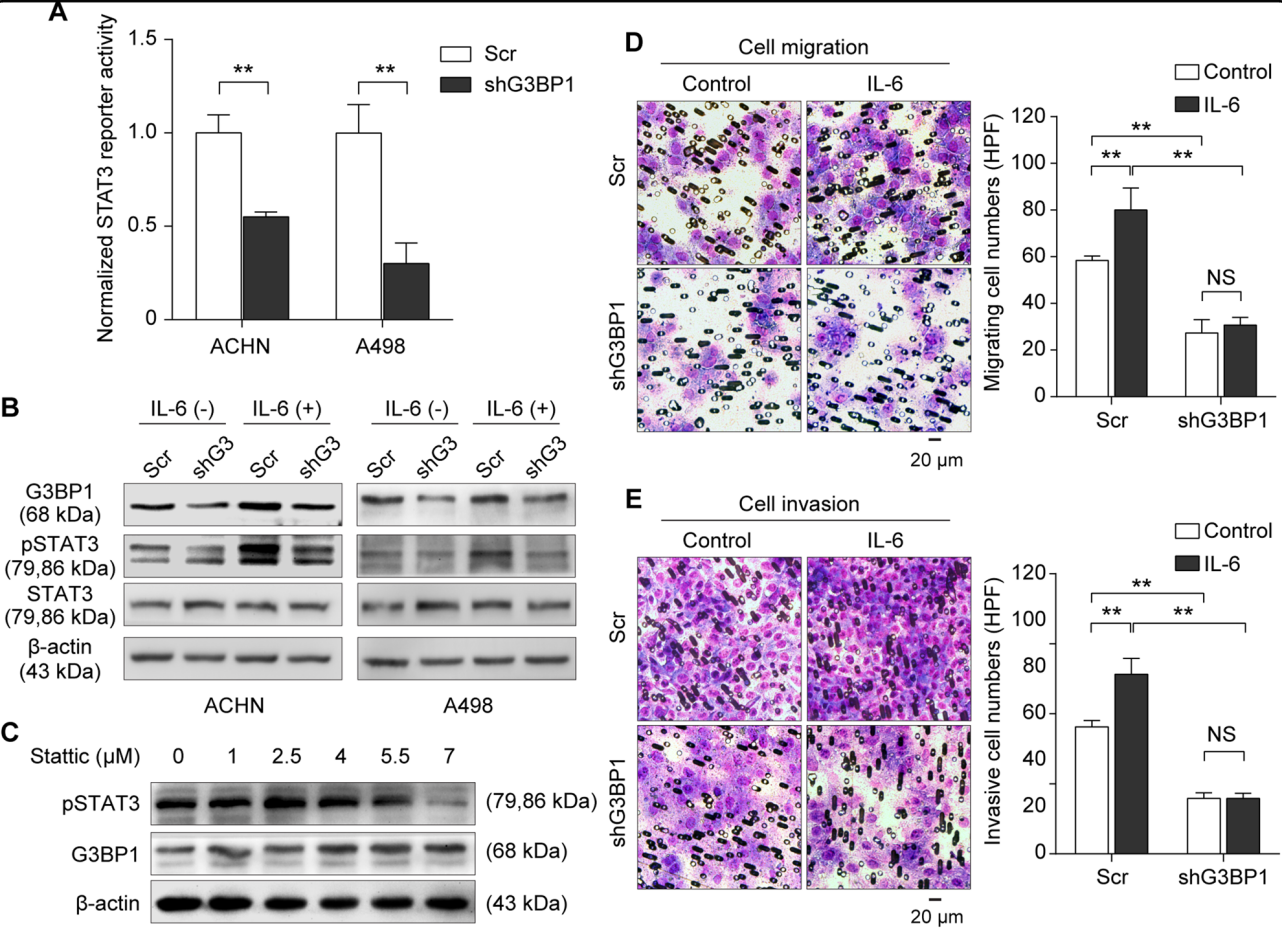
Knockdown of G3BP1 impaired IL-6-induced STAT3 activation and led to the suppression of RCC migration and invasion. **a** ACHN and A498 cells with stably knockdown of G3BP1 (shG3BP1) or scramble control (Scr) were co-transfected with STAT3 pathway firefly luciferase reporter (STAT3-luc) together with internal control Renilla luciferase reporter (pRL-TK) vector. Forty-eight hours after transfection, cell lysates were subjected to dual-luciferase assay. Data were obtained from three independent repeats and presented as mean ± s.d., **p < 0.01 by Student’s *t*-test. **b** ACHN cells with stably knockdown of G3BP1 (shG3) or scramble control (Scr) were treated with or without human recombinant IL-6 (100 ng/ml) for 48 h. The expressions of G3BP1, p-STAT3 (Tyr705), and total STAT3 were examined by Western blot. **c** ACHN cells were treated with STAT3 inhibitor stattic at the indicated concentration for 48 h, and expressions of G3BP1 and p-STAT3 (Tyr705) were determined by Western blot. **d**, **e** ACHN cells with stably knockdown of G3BP1 (shG3BP1) or scramble control (Scr) were treated with or without human recombinant IL-6 (100 ng/ml) for 48 h, and then (**d**) Transwell assay was performed to determine the cell migration and (**e**) Matrigel cell invasive assay was used to examine the cell invasion, respectively. Left panel: representative microscopic images. Right panel: quantitative analysis. Data were presented as mean ± s.d. from three independent experiments, and five random microscopic fields were acquired in each experiment for quantification, **p < 0.01 by one way ANOVA followed by Tukey’s post hoc test, NS: no significant difference.

To further explore the underlying molecular mechanism of G3BP1-mediated RCC EMT, we first searched for the oncogenic signaling pathways that can be affected by G3BP1 silencing, including STAT3, AP1, ISRE, TGFβ (p3TP), P53, NFAT, and WNT (TOPFlash)^36^ using pathway luciferase reporter system in RCC cells (Suppl Fig. 2). The results strongly suggested that G3BP1 knockdown caused significant impairments of multiple tumorigenic-related signaling pathways in RCC cells, including STAT3, AP1, and interferon (ISRE) signaling (Fig. 4a and Suppl Fig. 2). Evidence indicated that in response to extracellular insults, STAT3 is activated through tyrosine phosphorylation at Tyr705, and this constitutive activation of STAT3 is a pivotal event in RCC tumorigenesis and metastasis^37,38^. Further Western blotting results confirmed that G3BP1 silencing led to inhibition of STAT3 phosphorylation in RCC cells (Fig. 4b). Specifically, IL-6 is one of the well-documented cytokines which is abundantly expressed in RCC and enhances RCC cell proliferation and invasiveness through activation of STAT3 signaling^13,14^. In line with this notion, we further treated G3BP1 knockdown and scramble control cells with human recombinant IL-6 (100 ng/ml) for 48 h, and Western blotting results revealed that stimulation with IL-6 did not only activate STAT3 but also induced G3BP1 expression in scramble RCC cells. In contrast, IL-6-induced STAT3 phosphorylation was dramatically abolished in G3BP1 knockdown cells (Fig. 4b). In addition, we found that treatment with stattic, a specific inhibitor for STAT3 activation and dimerization, exhibited a profound effect on STAT3 phosphorylation, with no effect on the expression of G3BP1 in ACHN cells, suggesting STAT3 acts as a G3BP1 downstream signaling molecule (Fig. 4c). Together, the data firmly indicated that knockdown of G3BP1 blocked IL-6-induced STAT3 activation, and G3BP1 is a bridge molecule linking IL-6 and STAT3 signaling in RCC cells.

To verify the functional impact of the IL-6/G3BP1/STAT3 signaling axis in the regulation of EMT events in RCC, we next evaluated the effects of G3BP1 depletion on RCC cell migration and invasion with presence or absence of IL-6. The results revealed that treatment of IL-6 (100 ng/ml) significantly (*p* < 0.01) promoted tumor cell migration (Fig. 4d) and invasion (Fig. 4e) in RCC ACHN cells, while G3BP1 depletion strongly attenuated the IL-6-induced enhancement of RCC cell migration and invasion (Fig. 4d, e). Thus, these results functionally indicated that IL-6 increased RCC cell migrating and invasive abilities, which could be declined at least partially by the knockdown of G3BP1.

### Expression of G3BP1 is correlated with IL-6 and p-STAT3 in primary RCC patients

**Fig. 5:**
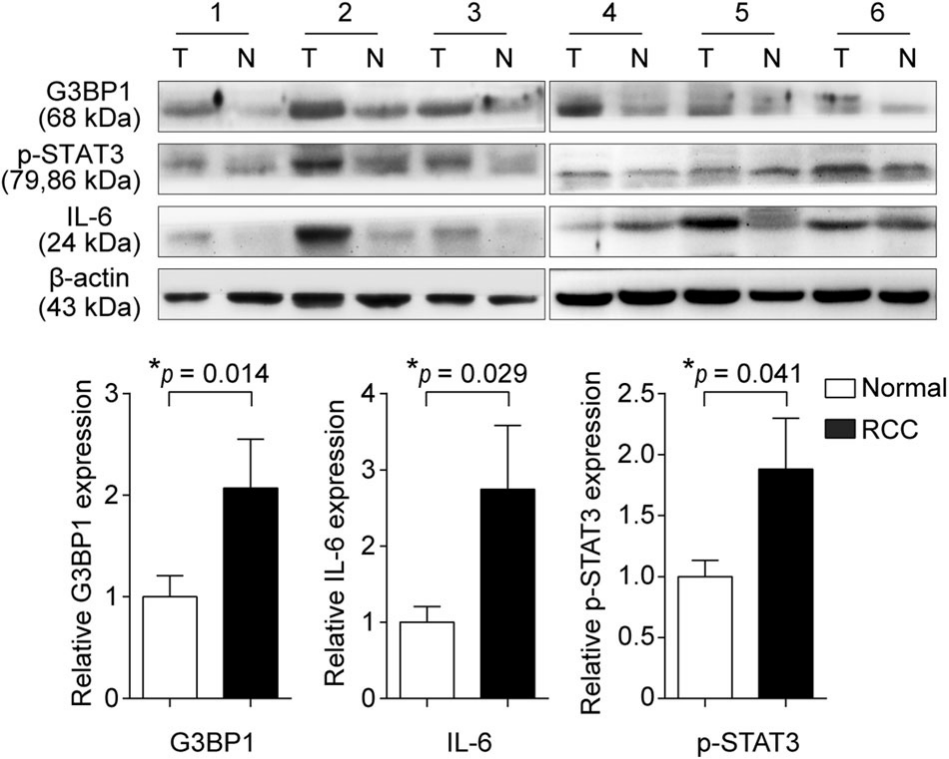
Expression of G3BP1 is correlated with IL-6 and p-STAT3 in primary RCC patients. The expressions of G3BP1, IL-6, and p-STAT3 (Tyr705) in a panel of 32 pairs of primary RCC (T) and adjacent normal kidney tissues (N) were examined by Western blot. Upper panel: representative results from six pairs of patient samples were shown. Lower panel: the expressions of G3BP1, p-STAT3 (Tyr705), and IL-6 were quantified by normalizing with β-actin, and all 32 pairs of RCC patient samples were analyzed using a paired *t*-test.

Given the significance of G3BP1 in controlling IL-6/G3BP1/STAT3 signaling in RCC migration and metastasis *in vitro*, we next address the correlations of G3BP1 with IL-6 and STAT3 activation in our clinical study cohort comprising of 32 pairs of primary RCC and their corresponding adjacent normal kidney tissues. Western blotting showed that the expressions of G3BP1, IL-6, and p-STAT3 were significantly (*p* < 0.05) elevated in RCC tissues as compared to those in the corresponding adjacent normal kidneys (Fig. 5). In addition, the expression of G3BP1 is positively correlated with IL-6 (*r* = 0.510, *p* = 0.003) and p-STAT3 (*r* = 0.683, *p* < 0.0001) in RCC tissues (Table 2), suggesting the associations of IL-6/G3BP1/STAT3 molecules in primary RCC.

**Table 2:** Correlation between G3BP1 and IL-6/pSTAT3 expressions in RCC

		G3BP1 expression		Spearman’s *r*	
		Up	Down	Correlation coefficient	*p* Value
IL-6	Up	21	2	0.510	0.003*
	Down	4	5		
pSTAT3	Up	20	0	0.683	<0.0001*
	Down	5	7		

Note: The expressions of G3BP1, IL-6 and pSTAT3 were quantified and compared between RCC tumor (T) and paired adjacent normal tissue (N). The relative ratio T/N > 1 was defined as “Up”, and T/N < 1 was defined as “Down”.

*p <  0.05 indicates statistical significance by Spearman’s correlation coefficient analysis.

### G3BP1 knockdown inhibited RCC tumor growth and metastasis *in vivo*

**Fig. 6:**
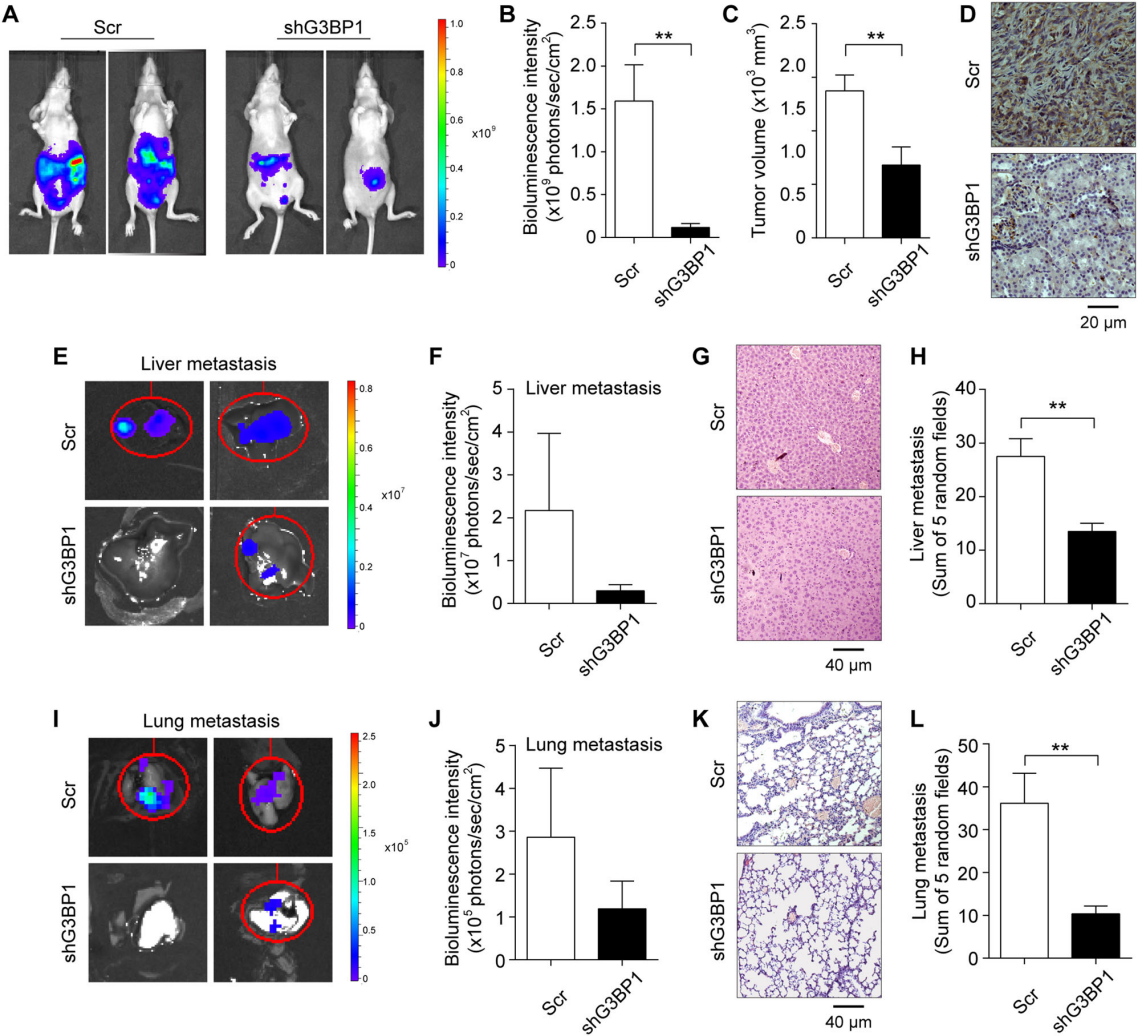
G3BP1 knockdown inhibited RCC tumor growth and metastasis *in vivo*. ACHN cells were transduced with Luciferase-labeled G3BP1 knockdown (shG3BP1-luc) or scramble control (Scr-luc) were used to implant into nude mice for 8 weeks, *n* = 6 each group. The bioluminescence was detected by IVIS imaging system, and followed by histological examination. **a** Tumor growth was examined by IVIS imaging system, and representative images were shown. **b** Tumor growth was quantified by bioluminescence intensity. **c** Primary tumor volume was measured and compared. **d** Mouse primary renal tumors were collected and processed for IHC staining of G3BP1, and representative images were shown. **e**–**l** Liver and lung metastases were examined by IVIS bioluminescence imaging system and histological H&E staining for detection of tumor foci. **e**, **i** Tumor metastases in liver (**e**) and lung (**i**) were examined by IVIS imaging system, and representative images were shown. **f**, **j** Quantified bioluminescence in liver (**f**) and lung (**j**) metastatic sites were calculated and compared. **g**, **k** Tumor metastatic foci were evaluated by H&E staining in liver (**g**) and lung (**k**), and representative images were shown. **h**, **l** Tumor metastatic foci in liver (**h**) and lung (**l**) were quantified and compared using five random views under the microscope. All data were presented as mean ± s.e.m, *n* = 6, **p < 0.01 by Student’s *t*-test.

To determine whether G3BP1 knockdown impacts RCC tumor growth and metastasis *in vivo*, we performed the orthotopic tumor xenografts with implantation of luciferase-labeled G3BP1 knockdown cells (ACHN-shG3BP1-luciferase) and control RCC cells (ACHN-Scr-luciferase) into the sub-renal capsule of adult nude mice left kidney. After 8 weeks, mice were intraperitoneally injected with luciferin and the bioluminescence intensity was detected and quantified for the primary tumors as well as lung and liver metastasis using the live bioluminescence IVIS imaging system. The results showed that a significant (*p* < 0.01) reduction of primary RCC tumor size was observed in mice with G3BP1 knockdown as compared to the scramble control group (Fig. 6a, b), suggesting G3BP1 plays an important role in RCC tumor growth *in vivo*. In addition, bioluminescent signals indicated that mice implanted with G3BP1 knockdown ACHN cells had much fewer liver and lung metastatic foci detected than those in scramble controls (Fig. 6e, f, i, j), thus revealing the functional role of G3BP1 in RCC metastasis.

Additionally, the primary RCC tumor and metastatic organs of liver and lung were isolated from the aforementioned mice. The primary renal tumor volumes were measured to be dramatically decreased in the G3BP1 knockdown group (Fig. 6c), and further IHC staining of the xenograft renal tissues confirmed the knockdown of G3BP1 expression in the ACHN-shG3BP1 group (Fig. 6d). Moreover, H&E staining of the excised livers and lungs clearly demonstrated that significantly (*p* < 0.01) more metastatic nodules were found in the scramble control group than the G3BP1 knockdown group (Fig. 6g, h, k, l). Collectively, the results from *in vivo* tumor xenografts model indicated that silencing of G3BP1 could suppress RCC tumor cell growth as well as inhibit RCC cell metastasis to liver and lung.

## Discussion

RCC frequently involves systemic metastasis which is the major cause of RCC mortality. However, the molecular basis of RCC progression remains elusive. In the present study, we revealed that the expression of G3BP1 is increased in primary RCC as compared to adjacent normal tissues, and G3BP1 is associated with higher levels of TNM stages and Fuhrman grade. Functionally, we found that knockdown of G3BP1 led to inhibition of tumor cell proliferation, migration, and invasion, as well as alterations of EMT markers using *in vitro* RCC cell line model. Consistently, our *in vivo* orthotopic tumor xenografts results also confirmed that knockdown of G3BP1 suppressed RCC tumor growth and metastasis in mice. Remarkably, we uncovered a novel signaling link that G3BP1 mechanistic connects upstream pro-inflammatory cytokine IL-6 stimulation to STAT3 activation, and eventually contributes to downstream cellular events, including promotion of RCC migration and metastasis. Together, our findings support a notion that G3BP1 promotes tumor progression and metastasis through IL-6/G3BP1/STAT3 signaling axis in RCC.

G3BP1, based on its crucial involvement in multiple biological processes and disease pathogenesis, including tumorigenesis, has emerged a target for molecular investigation in many oncology studies^24,26,29^. However, there is still limited knowledge about its correlation with progression of RCC. Overexpression of G3BP1 has been shown to promote breast cancer cells proliferation and induce EMT transition^26^. Also, knockdown of G3BP1 was reported to prevent tumor invasion and metastasis in xenograft model^26,29^. Recently, Dou *et al.* reported that upregulation of G3BP1 was associated with enhanced cell migration and poor survival of hepatocellular carcinoma (HCC), with no significant effect on HCC cell growth^29^. Similarly, our results demonstrated that G3BP1 was frequently upregulated at protein levels in primary RCC compared to adjacent normal tissues, and a higher level of G3BP1 expression is correlated with RCC progression. In addition, we analyzed but failed to detect the correlation of *G3BP1* mRNA expression and RCC survival using The Cancer Genome Altas (TCGA) database (data not shown). This suggests that G3BP1 might exert its tumorigenic function mainly at protein level, and the post-transcriptional regulation of G3BP1 might be crucial for its role in RCC. Indeed, it remains possible that the expression of G3BP1 displays a tissue-specific or cancer type-specific manner. Functionally, our results demonstrated that G3BP1 plays an important role in RCC cell proliferation, migration, invasion, and metastasis both *in vitro* and *in vivo*, suggesting that G3BP1 is a critical molecule in RCC progression. It is well established that the majority of RCC arise from renal tubule epithelial cells, and EMT is a key event that occurs during the invasion of cancers with an epithelial origin^38,39^. During EMT, cell*–-*cell and cell-matrix interactions are altered to facilitate tumor cell motility. In this content, the loose of adherens junction by “cadherin switching” is one of the hallmark events in EMT, which is characterized by the replacement of E-cadherin with N-cadherin^40^. We found that G3BP1 depletion is associated with upregulation of E-cadherin and downregulation of N-cadherin, as well as repression of mesenchymal marker vimentin, a type III intermediate filament related with cell-matrix interaction. Consistently, the expressions of E-cadherin repressors, Snail, and Slug were decreased in G3BP1 knockdown RCC cells. Our data clearly indicated that G3BP1 was indeed involved in regulating EMT in RCC. Besides the above-mentioned results from *in vitro* experiments, we also confirmed the G3BP1-mediated regulation of RCC tumor growth and metastasis using xenograft model *in vivo*. Taken together, G3BP1 is an important molecule not only involved in controlling tumor cell growth, but also in mediating EMT in RCC.

Previously, G3BP1 was reported to interact with the SH3 domain of RasGAP and modulate Ras signaling pathway^41^. Recently, Annibaldi *et al.* questioned G3BP1 being a genuine RasGAP-binding partner and suggested G3BP1-mediated signaling may not involve RasGAP^42^. In addition, G3BP1 has been shown to be involved in lung cancer cell migration and metastasis via Src/FAK signaling pathway^43^. In breast cancer, G3BP1 participated in the EMT and metastasis through regulating Smad signaling^26^. In our current study, we screened several oncogenic signaling pathways and identified STAT3, AP1, and type 1 interferon (ISRE) signaling pathways were dramatically impaired by a G3BP1 knockdown in RCC cells, indicating that G3BP1 is a pivotal joint molecule connecting multiple signaling pathways. It was also suggested that G3BP1-associated signaling pathways are diverse in cancers, and more importantly, to be cancer type specific. Consistently, we verified that both c-Myc and cyclin D1 as cell proliferation-associated downstream targets of AP1 signaling molecules repressed in G3BP1 knockdown RCC cells. In addition, to be of particular interest is the STAT3 signaling. STAT3 signaling pathway plays critical roles in tumor invasion and metastasis^38^. In response to extracellular signals, STAT3 is activated by phosphorylation of a conserved tyrosine residue and the phosphorylated STAT3 dimers undergo nuclear translocation where they regulate several “master” EMT transcriptional networks, including Snail and Slug^38,44^. Remarkably, our results showed that the depletion of G3BP1 inhibited STAT3 signaling, further suppressing the EMT phenotypes via transcriptional repression of Snail and Slug. This perhaps implies that G3BP1 functions through STAT3 signaling and eventually contribute to RCC EMT.

The crosstalk between tumor cells and their surrounding extracellular signals forms tumor microenvironment and leads to activation of oncogenic signaling pathways, including STAT3 signaling pathway. It was known that STAT3 can be robustly activated by IL-6 and EGF^13,14,45^. It was documented that EGF and its receptor EGFR are frequently overexpressed in many forms of RCC, and signaling cascade through EGF/EGFR/STAT3 play an important role in tumor cell growth and EMT in RCC^33,35,46^. In this study, we showed that the knockdown of G3BP1 dramatically impaired EGF-induced RCC cell chemotaxis, an essential component of tumor dissemination during progression and metastasis, indicating EGF-mediated chemotaxis in RCC is probably via G3BP1. Recent studies have shown that IL-6 can be produced by RCC cells, and enhanced levels of IL-6 increases the invasiveness of RCC^9–12,14^. Consistently, we demonstrated in the present study, IL-6 can activate STAT3 and lead to enhanced RCC cell migration and invasion. Importantly, we found that G3BP1 knockdown can significantly abolish the effect of IL-6 on STAT3 activation, as well as RCC cell migration and invasion, suggesting depletion of G3BP1 functionally blocks the IL-6/STAT3 signaling axis and diminishes the RCC metastatic potential. Importantly, we found that IL-6 itself can induce the G3BP1 expression in RCC cells. This further supports the notion that G3BP1 is a critical mediator that links IL-6 to STAT3 activation in RCC. In line with this finding, we showed the positive correlations between the expression of G3BP1 with IL-6 and p-STAT3 in our primary RCC cohort. Collectively, oncogenic G3BP1 serves as a signaling linkage molecule from upstream IL-6/EGF elicited stimulations to downstream STAT3 signaling, eventually contributing to promote tumor cell growth and metastasis in RCC.

In summary, the expression of G3BP1 is significantly higher in RCC comparing to para-tumor normal tissues, and knockdown of G3BP1 significantly decreased tumor cell growth and metastasis *in vitro* and *in vivo*. In addition, IL-6 can induce the expression of G3BP1, while G3BP1 depletion diminishes the IL-6-elicited STAT3 activation and leads to inhibition of tumor cell growth and invasion in RCC. It was well known that RCC is resistant to conventional radiotherapy and chemotherapy, and immunotherapies using a high dose of IL-2 and IFN-α are now applied for RCC treatment^47–50^. Unfortunately, clinical studies showed that the objective response rates are about 5–20% for RCC patients who use IL-2 or IFN-α treatment regimen^51,52^. Thus, there is a pressing need to develop new immunological targets for advanced treatment of RCC^14,53–56^. Recently, several antibodies developed for targeting blockage of the IL-6/STAT3 pathway for RCC immunotherapy have been registered for preclinical studies and phase I/II clinical trials^14,57^. Our findings suggest G3BP1 as a novel mediator of RCC tumor progression and further extends the current knowledge about IL-6/STAT3 associated mechanism in RCC carcinogenesis. Indeed, targeting the molecules of IL-6/G3BP1/STAT3 axis provides a novel and potential treatment strategy for RCC.

## Materials and methods

### Cell culture, transfection, and treatment

RCC cell lines including A498 and ACHN were obtained from American Type Culture Collection (ATCC). Cells were cultured in Eagle’s Minimum Essential Medium (MEM) (Gibco) supplemented with 10% FBS (HyClone) and 1× penicillin/streptomycin at 37 °C in the presence of 5% CO_2_. All cultures were routinely tested for absence of mycoplasma and for the retention of their respective morphology and growth characteristics. Specific short hairpin RNA (shRNA) was designed to target human G3BP1 and cloned into pLKO.1 lenti-vector, transfected into 293T cells together with lentivirus packaging plasmids, psAX2 and pMD2.G for 48 h using lipofectamine 2000 (Invitrogen). Lentivirus-containing medium was cleared by centrifugation and filtration with 0.45 µm filter and then added to culture medium of A498 and ACHN cells for shRNA transduction. G3BP1 stably knockdown (shG3BP1) and scramble control cells (Scr) were established with puromycin (10 μg/ml) selection for 10 days.

RCC cells were treated with 100 ng/ml of human recombinant IL-6 (Peprotech) for 48 h and subjected to cell migration and invasion assays while total proteins were extracted for Western blotting analysis. Stattic is a commercially available small molecule inhibitor of STAT3 activation and dimerization (Selleck). ACHN cells were seeded in six-well plate and treated with stattic (0, 1, 2.5, 4, 5.5, and 7 µM) for 48 h, and then total proteins were extracted for Western blotting analysis.

### Human patients

Human patient study cohort collected 43 pairs of primary RCC tissues and corresponding para-carcinoma normal tissues. All tissue samples were surgically removed and paraffin-embedded in the Tianjin Medical University Second Hospital from January 2012 to December 2015 with patients’ consents and ethical committee approval. Clinical parameters including age, gender, tumor size, histological type, and Fuhrman grade were collected. All patients had undergone radical nephrectomy or partial nephrectomy with no preoperative and postoperative adjuvant therapies. For RCC pathological classification, all samples were double-blind reviewed by two independent pathologists.

### Protein extraction and Western blot

Cells were harvested and lysed in SDS lysis buffer with 1× protease inhibitor cocktail and 1× phosphatase inhibitor cocktail (Roche). The concentration of total protein was determined by an Enhanced BCA Protein assay kit (Thermofisher Scientific). Equal amounts of each protein sample (40 μg) were separated by electrophoresis on SDS-PAGE and transferred onto polyvinylidene fluoride membranes (Invitrogen). After blocking with DifcoTM skim milk (Becton Dickinson), protein lysates were blotted with anti-G3BP1 antibody (Santa Cruz, SC), anti-pSTAT3 antibody (Cell Signaling Technology, CST), anti-STAT3 antibody (CST), anti-IL6 antibody (Saierbio), anti-E-cadherin antibody (SC), anti-Vimentin antibody (CST), anti-Snail antibody (CST), anti-Slug antibody (SC), anti-N-cadherin antibody (SC), anti-c-Myc antibody (SC), anti-cyclinD1, or anti-β-actin antibody (SC), overnight at 4 °C, and followed by incubation of corresponding horseradish peroxidase-conjugated anti-mouse or anti-rabbit secondary antibodies at room temperature for 1 h. The blots were visualized using ECL system (Millipore). Images were then analyzed and quantified with G:box (SYNGENE), and the expression of the target protein was normalized with the expression of internal control β-actin.

### IHC staining

Tissues were formalin-fixed and embedded in paraffin blocks and then cut into 5 µm thickness sections. Tissue sections on glass slides were deparaffinized in xylene, and dehydrated in gradient ethanol, followed by blocking of endogenous peroxidase activity in 3% hydrogen peroxide. Slides were then submerged in boiling citrate buffer for antigen retrieval. UltraVision Protein Block (BD) was applied to block non-specific background staining. The tissue sections were immunostained with an anti-G3BP1 antibody (Santa Cruz). After washing with PBS, the slides were immunoblotted using Max Vision HRP-Polymer anti-Rabbit IHC Kit (Miaxim.bio). Sections were developed by peroxidase substrate DAB Detection Kit (Miaxim.bio) and then counterstained by hematoxylin.

For the scoring system, all slides immunostained with G3BP1 antibody were scored by two independent pathologists for the percentage and intensity of cells showing specific immunostaining signals. The percentage score grading criteria of 0–5 were as follows: 0, (0–1)% of tumor cells positive; 1, (1–5)% of tumor cells were positive; 2, (6–10)% of cells were positive; 3, (11–20)% of cells were positive; 4, (21–50)% of cells were positive; and 5, >50% of cells were positive. The intensity of immunostaining level was determined by the subjective visual scoring of the brown stain as follows: 0, no staining; 1, yellow-brown staining; 2, brown staining. The final score was calculated by combining the percentage and intensity scores and recorded as negative (0–3) and positive (4–7)^30,31^.

### Cell proliferation assay

Cells (1 × 10^4^ per well) were plated in 96-well plates and cultured as described above. Cell proliferation was assessed at 24, 48, and 72 h after seeding using Cell Counting Kit-8 (CCK-8, Dojindo) according to the manufacturer’s protocol. Briefly, 10 μl of the CCK-8 solution was added to each well of the plate and incubated at 37 °C for 1 h. The absorbance of each well was measured at 450 nm using a microplate reader. All experiments were performed in triplicates.

### Cell migration assay

Cell migration was measured by transwell (8 μm pores, Corning) assay. Briefly, RCC cells (1 × 10^5^) were seeded into the upper chamber in serum-free medium, and medium containing 10% FBS was added to the lower chamber. After 24 h of incubation at 37 ℃, the cells attached to the lower surface of the membrane were fixed with 4% PFA and stained with hematoxylin. Cell numbers were counted in five randomly chosen fields under the microscope.

### Tumor cell invasion assay

Transwell insert (8 μm pores, Corning) were pre-coated with diluted growth factor-reduced matrigel (2.5 mg/ml, BD Biosciences). RCC cells (1 × 10^5^) resuspended in serum-free MEM were seeded into the upper chamber, and MEM medium containing 10% FBS were added to the lower chamber. The cells were allowed to invade through the membrane at 37 °C for 48 h. Non-migrating cells on the upper surface were removed, and only those cells attached to the lower surface of the membrane were fixed with 4% PFA and stained with hematoxylin, followed by counting in five randomly chosen fields under the microscope.

### Chemotaxis assay

Chemotaxis assay was performed using micro-Boyden chambers as described previously^58^. Briefly, RCC cells (2.5 × 10^4^) were seeded into the upper chambers in serum-free MEM, while 10% FBS and human recombinant EGF (Peprotech) with different concentrations were added to the lower chamber. The 8 μm of fibronectin-pretreated filter membrane was placed between two chambers. After 12 h incubation at 37 °C, the membrane was fixed and stained with hematoxylin. The number of migrating cells was counted in five randomly chosen fields under a light microscope.

### Pathway dual-luciferase reporter assay

The screening of signaling pathways by dual luciferase assay was carried out using a serial of pathway luciferase reporters (SABiosciences), including STAT3, AP1, ISRE, TGFβ (p3TP), P53, NFAT, and WNT (TOPFlash). RCC cells were co-transfected with pathway luciferase reporters and the internal control Renilla luciferase reporter (pRL-TK) vector using lipofectamine 2000. Forty-eight hours after transfection, cells were harvested and analyzed by the dual-luciferase assay kit (Promega). Each experiment was performed in triplicates, and at least three independent assays were conducted.

### Xenograft tumor growth and metastasis

Lentivirus-mediated luciferase-labeled G3BP1 stably knockdown (ACHN-shG3BP1-luciferase) and scramble control cells (ACHN-Scr-luciferase) were used. Nude mice (*n* = 6 each group, 6–8 weeks old, male:female = 1:1) were subjected to surgery with an incision in the back to exposure of the left kidney of mice. The mice were then injected with 1 × 10^6^ cells (log phase, mixed with Matrigel, 1:1) into the sub-renal capsule and incision closed. Eight weeks later, mice were intraperitoneally injected with luciferin (30 µg per mouse), followed by bioluminescence detection for primary tumors and metastasis in liver and lung using the live IVIS imaging system (Perkin Elmer). The mice were then sacrificed, and primary RCC tumors were isolated. Tumors were measured by largest and smallest diameters to calculate tumor volume. Primary RCC tumors, livers, and lungs were also fixed in formalin and embedded in paraffin. Serial sections and H&E staining were then performed to examine metastasis.

### Statistical analysis

All data were presented as the mean ± s.d. Differences in mean values between two groups were analyzed by *t*-test. Differences in mean values among multiple groups and/or multiple conditions were analyzed by one-way ANOVA or two-way ANOVA, followed by post hoc test. *χ*^2^ test was used for statistical analysis of the correlations between G3BP1 expression and clinicopathologic parameters. The correlations between the expressions of G3BP1, IL-6, and p-STAT3 were analyzed using Spearman test. Differences with *p < 0.05 were considered as statistically significant.

## Electronic supplementary material

Supplementary Figure Legend

Supplemental Figure 1

Supplemental Figure 2
