# Supplementary material for: G3BP1 promotes tumor progression and metastasis through IL-6/G3BP1/STAT3 signaling axis in renal cell carcinomas
Source: Cell Death Dis. 2018 May 2;9(5):501. doi: 10.1038/s41419-018-0504-2 (PMC5931548; doi:10.1038/s41419-018-0504-2)
Supplement: Supplementary file 1 — Supplementary Figure Legend [file 41419_2018_504_MOESM1_ESM.doc]

**Supplementary material**

**Suppl. Fig. 1. G3BP1 knockdown in RCC cells.** ACHN and A498 cells were transduced with lentivirus mediated G3BP1-specific shRNA (shG3BP1) or scramble control (Scr), and the efficiency of G3BP1 knockdown was examined by (A) qRT-PCR; (B) Western blotting. ** *p*<0.01 by Student’s *t*-test.

**Suppl. Fig. 2.** **Knockdown of G3BP1 impaired multiple signaling pathways in RCC.** ACHN cells with stably knockdown of G3BP1 (shG3BP1) or scramble control (Scr) were co-transfected with oncogenic signaling pathway reporters, including pSTAT3, pAP1, pISRE, p3TP (TGFβ), pP53, pNFAT and TOPFlash (WNT), together with internal control Renilla luciferase reporter pRL-TK vector. Forty-eight hours after transfection, cell lysates were subjected to dual-luciferase assay. Data were obtained from 3 independent repeats and presented as mean ± s.d., ** *p*<0.01 by Student’s *t*-test.
