# Supplementary figures and images for: G3BP1 promotes tumor progression and metastasis through IL-6/G3BP1/STAT3 signaling axis in renal cell carcinomas
Source: Cell Death Dis. 2018 May 2;9(5):501. doi: 10.1038/s41419-018-0504-2 (PMC5931548; doi:10.1038/s41419-018-0504-2)

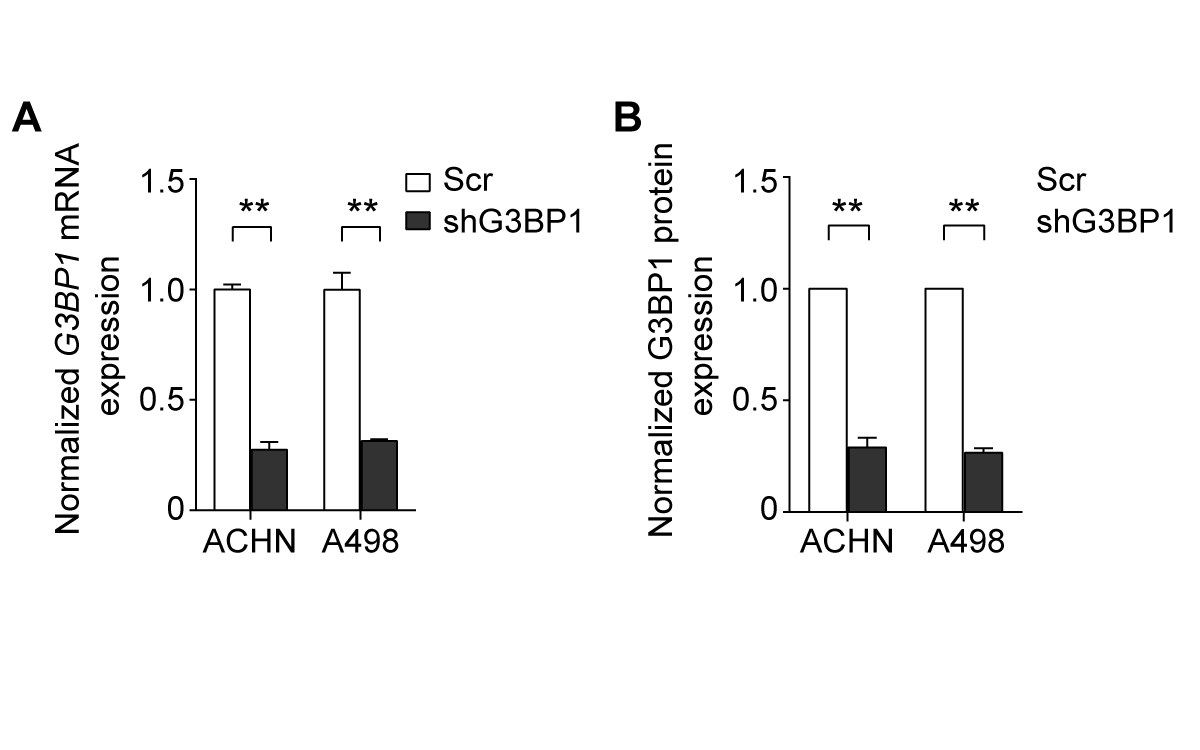

Supplement: Supplementary file 2 — Supplemental Figure 1 [file 41419_2018_504_MOESM2_ESM.tif]

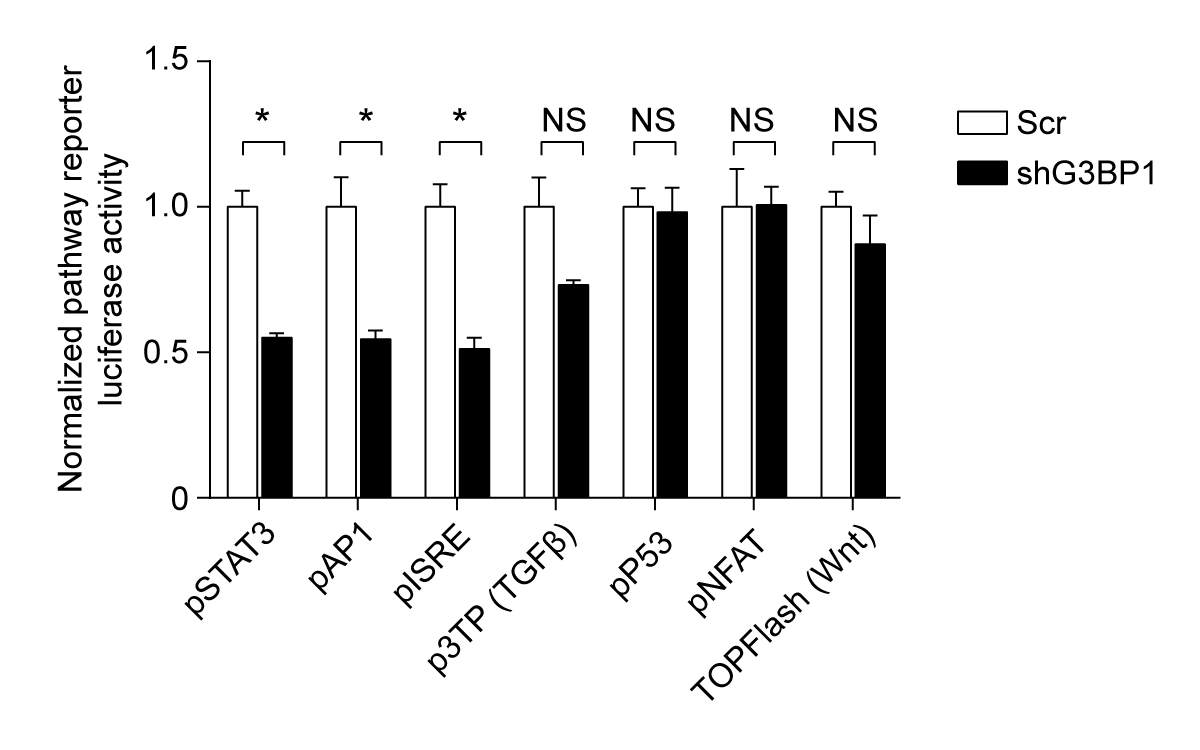

Supplement: Supplementary file 3 — Supplemental Figure 2 [file 41419_2018_504_MOESM3_ESM.tif]
